# 

**Published:** 1882-05

**Authors:** 


### *TABLE OF CONTENTS FOR MAY, 1882.*

ORIGINAL COMMUNICATIONS. PAGE

ART. I.—The Treatment of Diphtheria. By J. H. CARSTENS, M.D. ........289

CLINICAL REPORTS.

ART. II.—Acute Lobar Pneumonia with Cardiac Failure. By CHARLES W. PILGRIM, M.D. ........292
ART. III.—Aneurism of the Innominate Artery. By J. S. LYNCH, M.D. .....295
CORRESPONDENCE ........299

ORIGINAL TRANSLATIONS AND ABSTRACTS.

The Prognosis and Treatment of Ankle-Joint Disease ........300
Is there a Bacillus Lepræ? ........304
The Influence of Operations upon the Prolongation of Life and Permanent Recovery in Carcinoma of the Breast ........308
Is the Ovarian Cell Pathognomonic? ........313
Delirium Produced by Salicylic Acid ........314
Vicarious Menstruation ........314
Borax and Boracic Acid in Otorrhœa ........314
Characteristics of Saliva in Syphilitics ........315

SELECTIONS.

Emulsions, and how to make them ........320
What is Tubercle? ........324
A Simple Way of Performing Optico-ciliary Neurotomy—The Proposed Substitute for Enucleation ........327
Mercury and Other Remedies in the Treatment of Syphilis ........328
The Prophylaxis of Erysipelas ........329
Cyst of the Pancreas ........329
Constipation in Infants ........330
Treatment of Hernias of Long Standing ........330
Saline Arterial Injections in Collapse ........331
Moral Insanity ........332
Arsenical Paralysis ........332
Filaria Sanguinis Hominis ........333
The Sensation of Thirst ........334
Osseous Tissue Formed from Transplanted Bone-Marrow ........335
Skin-Grafting, our Present Knowledge ........336

EDITORIAL DEPARTMENT.

The Code of Ethics of the New York State Medical Society and the American Medical Association ........338
Are we Slaves or Freemen? ........340
Is Deafness Increasing? ........341
Resignation of the Post-Graduate Faculty of the Medical Department of the University of the City of New York ........341
CURRENT NEWS AND OPINION ........342–345
REVIEWS AND BIBLIOGRAPHICAL NOTICES ........346–348

POPULAR SCIENCE DEPARTMENT.

A Discovery of a City of Cliff Dwellers ........349
Morning Drams ........351
The Sandias—Past and Present ........352
Photo-Printing in Colors ........352
The Greenland Glaciers ........353
A Scientific Smuggler ........354
Birth and Breeding ........355

